# Infants' Prefrontal Hemodynamic Responses and Functional Connectivity During Joint Attention in an Interactive-Live Setting

**DOI:** 10.3389/fmedt.2022.821248

**Published:** 2022-06-15

**Authors:** Nozomi Naoi, Yasuyo Minagawa, Jun-ichi Yamamoto, Shozo Kojima

**Affiliations:** ^1^Department of Psychology and Linguistics, International Christian University, Tokyo, Japan; ^2^Department of Psychology, Faculty of Letters, Keio University, Yokohama, Japan; ^3^Graduate School of Human Relations, Keio University, Tokyo, Japan; ^4^Core Research for Evolutional Science and Technology (CREST), Japan Science and Technology Agency, Tokyo, Japan

**Keywords:** hemodynamic responses, cerebral, Responding to Joint Attention, Initiating Joint Attention, functional connectivity, interactive-live paradigm, infants, NIRS

## Abstract

The present study examined cerebral hemodynamic responses and functional connectivity during joint attention either initiated by infants (Initiating Joint Attention, IJA condition) or by their partner (Responding to Joint Attention, RJA condition). To capture responses to natural social cues in infants aged 7–12 months using functional near-infrared spectroscopy (fNIRS), we employed an interactive-live paradigm for IJA and RJA. During the measurement, an adult sat facing an infant, and objects, such as small stuffed animals, paired with sound toys were presented to the right or left side of the screen. In the RJA condition, the adult gazed at the infants' eyes and then to the objects to encourage the infants to follow the adult's gaze. On the other hand, in the IJA condition, the adult followed the infant's gaze as it shifted to the presented object. Our results indicate that the concentration of oxy-Hb in the bilateral ventral prefrontal region had significantly decreased, then followed by an increase in the right dorsal prefrontal region in the RJA. In addition, a selective activation in the bilateral dorsal prefrontal region was seen in the IJA condition. Moreover, the infants exhibited increased functional connectivity especially within the right ventral prefrontal region during RJA condition when compared with IJA conditions. These findings suggest that RJA and IJA recruit specific brain networks localized in the prefrontal cortex of infants.

## Introduction

Before language acquisition, infants develop the ability to communicate with others through non-verbal means called “joint attention” (1). Joint attention refers to the ability of individuals to coordinate visual attention with others on a given object or an event (1–3).

In adult-infant interactions, joint attention can be established either by Responding to Joint Attention (RJA) or by Initiating Joint Attention (IJA). During an episode of RJA, infants respond to adults' attempts to call the infants' attention to an object using prompts such as pointing, turning their head, or shifting their gaze. In the typical course of development, infants' response to an adult's joint attention cues emerges between 6 and 12 months (4). During an episode of IJA, the infant uses similar directional signals including gaze shifting and pointing to an object or an event to initiate coordinated attention with others (5–7). The infants' ability to spontaneously shift their gaze and/or point is expected to appear around 9–12 months in typical development (2).

Many studies suggest that joint attention play important roles in a child's social, cognitive, and language development [e.g., (8–13)]. For example, individual differences in RJA skills at 12 months and IJA skills at 18 months predict subsequent language skills at 24 months (13).

However, the neural processes underlying joint attention functions have not been fully examined. Redcay et al. (14) examined the neural correlates of IJA and RJA in adults using a functional magnetic resonance imaging (fMRI). During fMRI measurements, the participant and experimenter participated in a face-to-face real-time game using a live video feed to find targets together. During RJA conditions, participants were presented with the experimenter's face and four “mouse houses” connected by pipes surrounding their face on the monitor. Then they were asked to follow the experimenter's gaze to a target (hidden mouse) located in one of the four “mouse houses”. For IJA, participants were asked to shift their gaze to a particular location and then to cue the experimenter to look there. In the control condition, the participants were asked to shift their gaze to a target while the experimenter's eyes closed.

Redcay et al. (14) demonstrated that a greater response in the dorsal medial prefrontal cortex (dmPFC) and right posterior superior temporal sulcus (STS) occurs during both IJA and RJA when compared with the control condition. They used the control condition in which the participant shifted their gaze while the experimenter did not shift her gaze. Therefore, activations due to participants' gaze shifting were subtracted out from those in IJA and RJA conditions. The results suggested that the dmPFC is associated with the coordination of attention during triadic social interactions; to include the self, the other, and the object, which is commonly required for both IJA and RJA conditions (15–18). Additionally, previous fMRI studies revealed greater activation in the posterior STS during the tasks involving the coordination of attention through gaze shift cues, which is also a common neural substrate for both IJA and RJA conditions (19, 20). Therefore, RJA and IJA recruit common brain regions such as the dmPFC and STS during joint attention, which is either initiated by participants or by their experimenter. In addition, Redcay et al. (14) also reported brain regions that are specifically involved in RJA or IJA. When compared to the control condition, the participants exhibited a greater response in the ventromedial prefrontal cortex (vmPFC) in RJA conditions but not in IJA conditions. RJA requires greater coordinated attention to another's gaze shift than IJA. On the other hand, IJA requires greater coordination of attention between the participant and object than RJA. For IJA, the intraparietal sulcus and middle frontal gyrus (MFG) exhibited enhanced activation. Therefore, distinct brain regions were also engaged during joint attention whether initiated by participants or by their experimenter.

To date, neural substrates of joint attention have been rarely examined in infants. Striano et al. (21) developed an interactive-live paradigm to assess the neural mechanisms of RJA in 9-month-old infants. They used Event Related Potentials (ERPs) to measure infants' electrical brain activity. Under joint attention conditions, an adult gazed at the infant's face and then shifted gaze to the monitor displaying an object. In the no-joint attention condition, the adult gazed only at the object presented on the monitor without looking at the infant's face. Striano et al. (21) identified a negative component (referred to as Nc) of the infant ERPs, indexes attention orientation. They observed enhanced Nc when infants viewed the objects in the joint attention condition relative to the no-joint attention condition. Striano et al. (21) suggested that infants give more attention to objects when prompted by the adult's gaze shift. However, this ERP study could not reveal any functional localization of joint attention in infants due to its limited spatial resolution.

Using functional near-infrared spectroscopy (fNIRS), which has higher spatial resolution then ERPs, Grossmann and Johnson (22) examined 5-month-old infants' frontal and temporal cortex responses during social interactions. For the joint attention condition, a virtual agent presented on the monitor looked and turned toward an object presented either to the left or to the right side of the agent's face. For the no-referent condition, the agent looked and turned toward the side where there was no object. In the no eye contact condition, the agent looked at the object without establishing any eye contact with the infant. The results demonstrate significantly increased activation in the left dorsal prefrontal cortex when engaged in joint attention. Furthermore, Grossmann et al. (23) compared 5-month-old infants' brain activation under two conditions. One involved the virtual agent following the infants' gaze to an object. For the second condition, the virtual agent only looked at an object which the infant had not looked at previously. The results identified a significantly increased activation of the left prefrontal area when the agent followed the infants' gaze to an object compared to baseline, but there was no significant difference in brain activity between the two experimental conditions. The results of these studies suggest the involvement of the left prefrontal cortex both when joint attention episodes were initiated by virtual agents (22) and by infants (23). Grossmann et al. (23) also discussed that left prefrontal activity reflects motivation to approach a social partner. These findings may be influenced by the age of the infants participating in these previous studies. As mentioned above, in typical development, following the gaze of others occurs reliably from around 6 months of age (4). In addition, the initiation of joint attention begins to occur at around 9 months of age (2). Therefore, 5 months of age is the age when joint attention does not yet occur in behavior. Furthermore, in the early development of joint attention, it has been suggested that joint attention between a caregiver and a child is mainly maintained by the caregiver, which is called Supported Joint Attention [SJA, (24)]. In this stage of SJA, infants may be less sensitive to whether joint attention episodes are initiated by themselves or by others. Therefore, in this study, we focused on infants who were old enough to initiate and respond to joint attention in their behavior.

The results of these previous studies provided some evidence for frontal brain region involvement in joint attention for both adults and infants. However, the underlying neural substrates in infants which respond to and initiate joint attention have not been fully examined for natural adult-infant interactions. In previous studies of infants, a virtual agent on a monitor acted as the infant's social partner, and an object, such as a picture of a car, presented on the monitor was the target of joint attention (22, 23). In these studies, infants' visual attention to the target stimulus presented on the monitor was induced exogenously by moving stimuli or by highlighting them with a red frame. Thus, under these experimental conditions, it was difficult to assess whether infants' gaze shifts were caused by an endogenous motivation to share visual attention directed at the target with others. The functions of infants' IJA have been extensively examined, and previous behavioral studies have suggested that the child's initiation of gaze shift between an object and a social partner or initiation of pointing during a joint attention episode have a “declarative” function which directs adult attention to the child's object of interest rather than an “imperative” function which is the function of requesting the object (25–27). In other words, IJA can be defined as the act of infants directing their visual attention to an object of interest, checking the adult's gaze, and then (if the adult is not gazing at the target) moving their gaze to the target again to direct the adult's attention to the object. However, previous infant fNIRS studies did not evaluate whether infants check the gaze of the virtual agent presented on the monitor before or after their initial gaze toward the object.

Additionally, previous infant studies have demonstrated that infants' responses to the person presented in the monitor and a real person differ. For example, Barr and Hayne (28) compared the performance of delayed imitation in 12-, 15-, and 18-month-old infants 24 h after they observed a model of a specific action presented by a real person or video. The results demonstrated that the performance of delayed imitation was higher in the group that observed models of a real person than a video person in all age groups. Furthermore, in a study of phonological discrimination in younger children (9 months), comparing infants in an English-speaking environment who were spoken to and interacted with a native Mandarin speaker in person with those who were shown the same sessions on video. The group that experienced the interaction with a real person showed a superior increased ability in a Mandarin phonological discrimination task (29). In other words, infants' social responsiveness has been shown to be higher to real people than to people presented on a monitor. Therefore, it is important to examine infants' brain responses during joint attention using more natural live settings with real persons as joint attention partners and real objects as joint attention targets.

In previous studies examining the neural networks associated with joint attention in adults based on brain functional connectivity in the resting state, the involvement of multiple different neural networks has been suggested. One is a network related to social cognition that includes the medial prefrontal cortex (mPFC) and the STS. The mPFC is also a part of the default mode network, which has been implicated in self-referential functions and endogenous shifts of attention (14, 30). Another neural network associated with joint attention is the frontoparietal attention network. The dorsolateral frontoparietal or dorsal attention network is associated with voluntary shifts of visual-spatial attention based on internal goals (31, 32). The ventral frontal or ventral attention network contributes to the detection of salient stimuli in the environment and is also referred to as the saliency network (31). These two attentional networks have different dominance in the hemispheric laterality. The dorsal attention network is a bilateral network, while the ventral attention network is a right-lateralized network (31). Therefore, it has been suggested that multiple different functional networks are associated with joint attention in adults and that different regions of the frontal area contribute to each functional network.

To date, few studies have examined functional brain networks associated with joint attention in infants. Therefore, we also focused on the functional connectivity of infants during joint attention. Eggebrecht et al. (33) used resting-state functional connectivity MRI (fcMRI) (34) to examine the relationship between functional connectivity of the brain and the development of the IJA in 12- and 24-month-old infants and toddlers. The results suggested that functional connectivity between the visual network and dorsal attention network, as well as the visual network and the posterior cingulate area in the default mode network, are associated with the development of joint attention at 12 months. Furthermore, at 24 months of age, functional connectivity between the frontoparietal and dorsal attention networks, the saliency network and the anterior part of default mode network, and the frontoparietal attentional networks were associated with the development of joint attention. Thus, while increased brain activities in the left prefrontal regions specific to joint attention have been reported in infants as young as 12 months (22, 23), there is no evidence of functional connectivity in the social cognitive or default mode networks, and dorsal or ventral frontoparietal attentional networks, as seen in adult participants relating to joint attention. Eggebrecht et al. (33) examined the relationship between brain functional connectivity in infants while in a sleeping state and behavioral development of IJA measured in infants in a wakened state but did not directly examine the functional networks during natural joint attentional interactions.

Therefore, in the present study, we examined functional connectivity within the frontal lobe during natural joint attentional interactions between infants and an adult. Previous MRI studies in adults and infants have suggested that the multiple functional networks associated with joint attention involve a wide range of brain regions, not limited to the frontal lobe (14, 33). However, because this study used fNIRS to examine the frontal regions of infants, it is not possible to directly examine functional networks involving brain regions other than frontal regions and subcortical regions. On the other hand, previous MRI studies have suggested that different frontal regions contribute to different functional networks (14, 33). Therefore, the present study exploratively examined how infants' functional brain connectivity within the frontal region during joint attention episodes differs between IJA and RJA conditions.

The present study examined cerebral hemodynamic response over the prefrontal region during joint attention episodes in infants aged 7–12 months using fNIRS. We used an interactive-live paradigm which elicited robust RJA and IJA in infants. This study directly examined cerebral hemodynamic responses during RJA and IJA, as compared to the baseline, in which infants were shown a toy without eye contact.

Based on previous studies in infants (22) and adults (14), we expected increased activity in the dorsal frontal area during both the IJA and RJA conditions. Furthermore, unlike Grossmann and Johnson (22) and Grossmann et al. (23), our present study was conducted with infants who demonstrated IJA and RJA in behavior. We predicted that a direct comparison of the RJA and IJA conditions would reveal brain regions specific to each condition. When compared to the IJA condition, we expected to find higher levels of activation in ventral prefrontal region in RJA condition, which was shown in fMRI studies in adult participants (14). We also predicted that the IJA condition would show a higher level of activity in the middle frontal regions compared to the RJA condition, which was also shown in fMRI studies in adult participants (14). For functional connectivity within the frontal lobe, we predicted that stronger functional connectivity would be found within the right prefrontal regions in the RJA condition compared to the IJA condition, because RJA is more associated with the ventral attentional network which is right-lateralized. On the other hand, because IJA is more associated with a dorsal attentional network, we predicted that there would be a stronger functional connectivity between bilateral dorsal prefrontal regions in the IJA condition compared to the RJA condition.

## Materials and Methods

### Participants

A total of 22 infants ranging in age from 7 to 12 months (mean age: 9.5 months, 8 male infants) and their mothers participated in this study. Participants were recruited as paid participants from local advertisements. At the time of recruitment, all infants were pre-screened to determine whether or not they exhibited any developmental delays using the Kyoto Scale of Psychological Development [KSPD, (35)]. The present study was carried out with the informed consent of the parents. The study was conducted with the approval of the ethics committee of Keio University, Faculty of Literature (No. 04001).

### Setting

**Figure 1:**
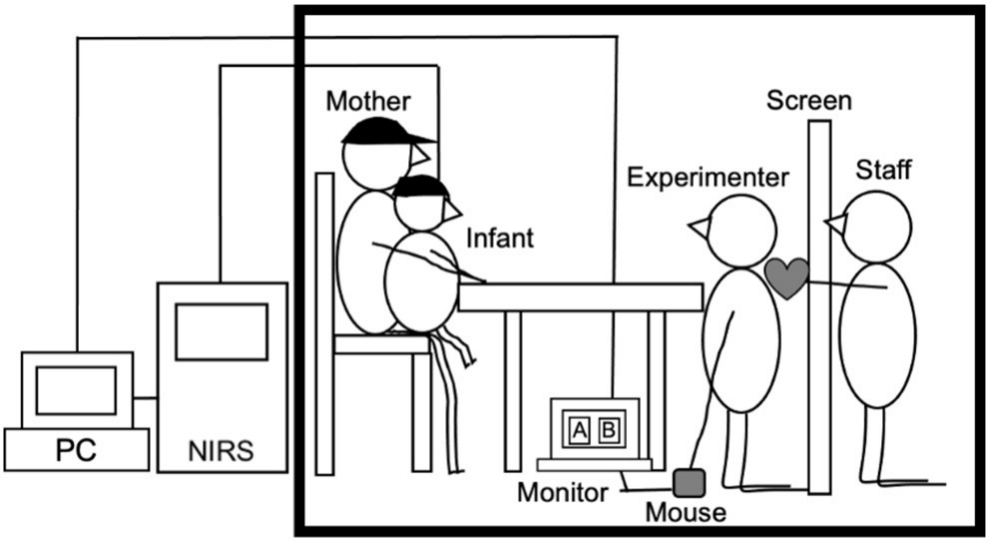
Experimental settings.

Measurements took place in a 4.5 × 3.5 m sound attenuated room at the University (see Figure 1). Infants sat on their mother's lap, while a female experimenter played the role of social partner for infants. She sat facing the child on her knees, such that she was approximately at the child's eye level, 40 cm away from the child. The experimenter placed a response recording sheet with a predetermined order of trials for each participant under the table and recorded the infant's behavior, such as crying and fussiness, on this sheet. Another staff member was hidden behind a screen (0.9 × 1.8 m) positioned 60 cm from the infant. During all trials, the staff presented different stimuli to the right or left side of the screen. The stimuli consisted of six different stuffed animals and dolls (size 5 × 10 cm−10 × 12 cm) that were paired with one of two kinds of sound toys (a maraca or a baby rattle). In addition, there were toys such as a pull-string-fan toy on the table that made very little sound. These toys were used to maintain the infant's attention during the baseline. In addition, mothers wore sun visors that prevented them from looking at the target object during the experiment.

### Procedures

**Figure 2:**
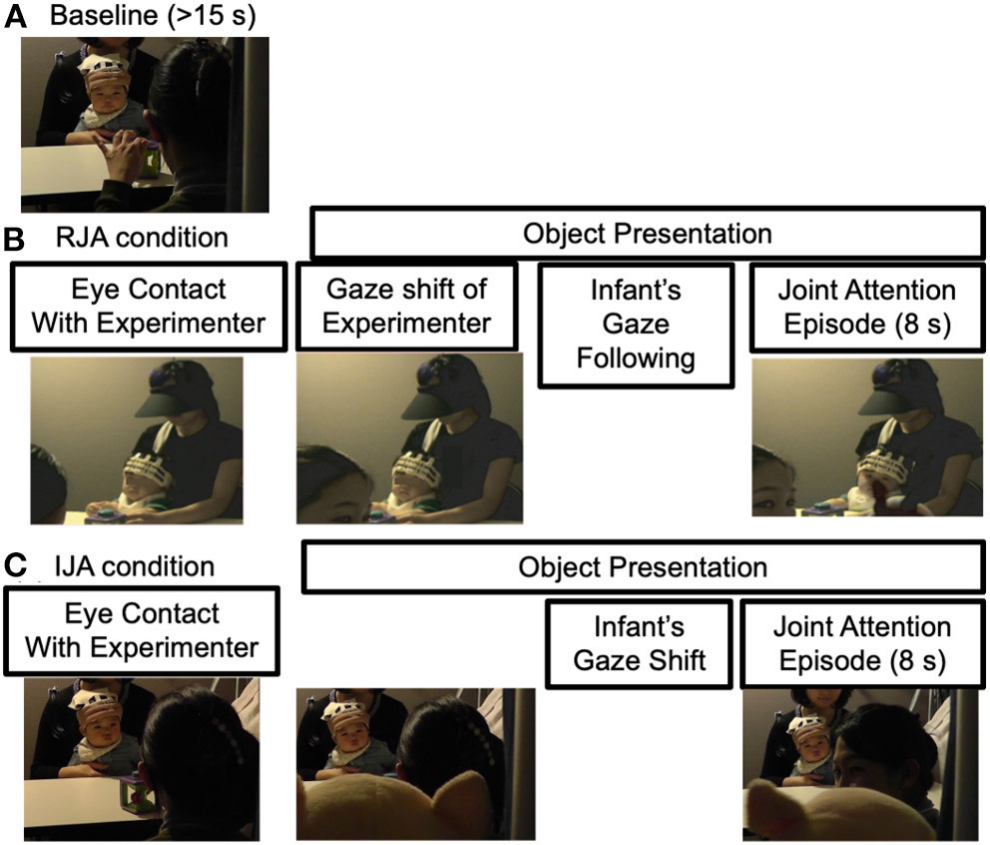
Experimental protocol during **(A)** the baseline, **(B)** RJA condition, and **(C)** IJA condition.

During a measurement session, a baseline period was administered at the beginning of each measurement. Each experimental condition (RJA or IJA) was alternated with a baseline period with an additional one at the end (see Figure 2).

Throughout the baseline, the experimenter activated the toy on the table out of reach but within view of the infant for a minimum of 15 s. During this period, the experimenter did not interact with the infant through eye contact. After the minimum of 15 s had passed, one of the two experimental conditions started.

Under the table, there was a monitor displaying “A” and “B” buttons assigned for each condition. The experimenter clicked one of the two buttons using a mouse to record “ON” event markers into the NIRS recordings. These buttons were by default colored blue and turned yellow when they were on. Although the staff was hidden behind a screen, this screen was made of cloth and the staff could see the buttons presented on the monitor through the screen. On the other hand, neither the staff behind the screen nor the monitor was visible to the infants or mothers. When either button was on, the staff behind the screen presented stimuli to the right or left of the screen 5 s later. The stuffed animals and dolls functioned as a visual target of joint attention, and this was paired with the sound toy to make sure the infants' attention was attracted. The stimuli were presented to the infant so that the stuffed animal or doll appeared to be holding and moving the sound toy. The staff was not informed of which experimental condition was assigned to which buttons.

For the RJA condition, the experimenter clicked on the button to place the “ON” event marker. The experimenter gazed at the infants' eyes and then looked back to the stimulus objects saying “Ah!” Then, the staff behind the screen presented the stimuli just above the experimenter's left or right shoulder. The experimenter went on looking at the presented stimuli to maintain the joint attention episode for 8 s, and then she looked at the infant's face again, saying “That's a doll!”. Then the experimenter clicked on the button again to place the “OFF” event marker and the staff hid the stimuli behind the screen.

For the IJA condition, following the experimenter's clicking on the button to place the “ON” event marker, the experimenter gazed at the infants' eyes and the staff behind the screen presented the stimuli in exactly the same manner as the RJA condition. The experimenter remained still and silent and waited ~10 s to determine whether the infant would initiate joint attention to the presented stimuli. If an infant initiated a gaze shift from the target stimuli to the experimenter's eyes and back again during target presentation, then the experimenter immediately responded to the child by looking at the target stimuli saying “Ah!” She went on looking at the presented stimuli to maintain the joint attention episode for an additional 8 s. Then the experimenter looked at the infant's face again, saying “That's a doll!” Then the experimenter clicked on the button again to place the “OFF” event marker and the staff hid the stimulus objects behind the screen.

The number of adult vocalizations and the degree of positive affirmations were adjusted to be the same in both experimental conditions. The RJA and IJA trials were presented to each participant in a predetermined pseudo-randomized order so that no more than two of any given experimental trials were presented consecutively. In addition, the stimuli were presented for no more than two consecutive trials on the left or right side of the screen. Both RJA and IJA trials were repeated at least 5 times. Along with the same lines, the first trial was counterbalanced among participants with RJA or IJA trials. A minimum of 10 trials were obtained (5 trials for each of the RJA and IJA conditions, and 5 trials on the right and left sides). The measurement was terminated when the infant became too bored or fussy, making it difficult to continue the measurement, or when neither RJA nor IJA occurred for five or more consecutive trials. The experiment was also terminated when a maximum of eight trials were completed in each experimental condition.

### NIRS Measurement

**Figure 3:**
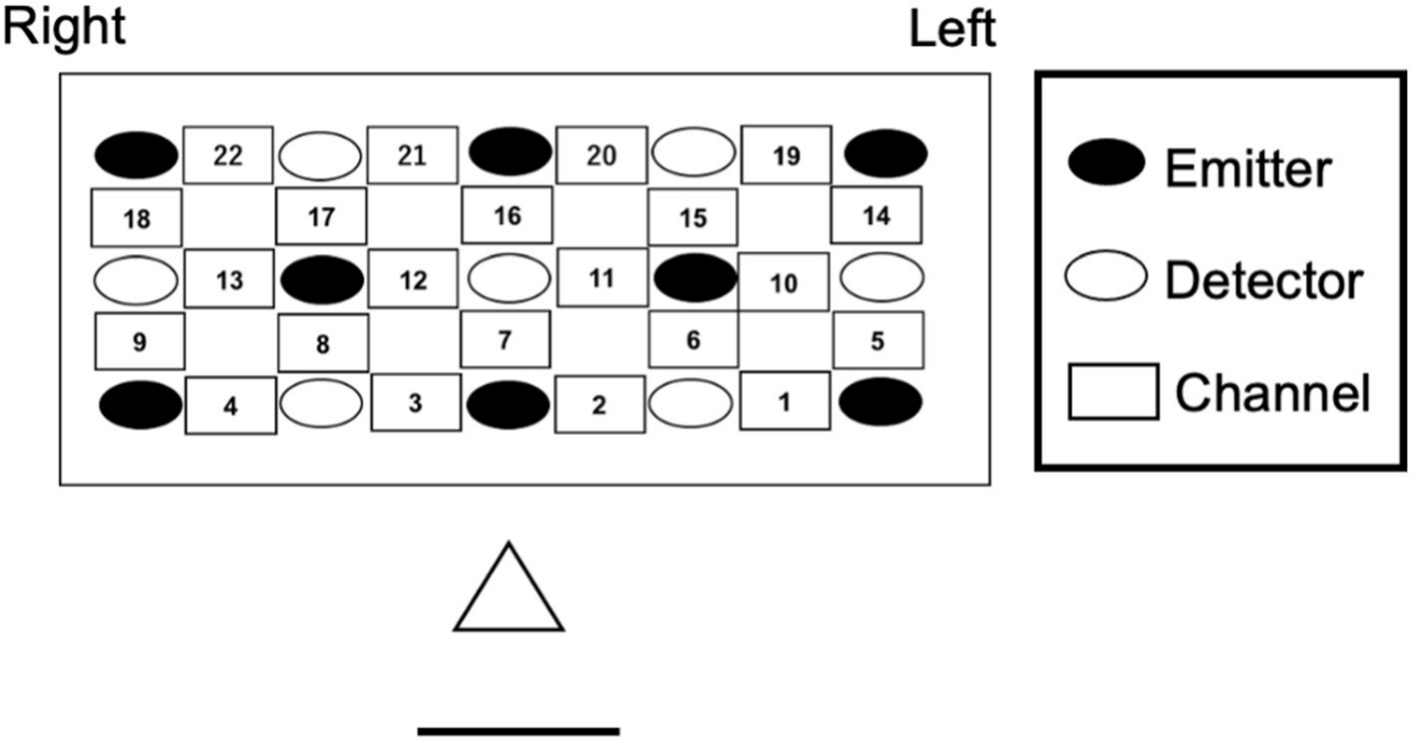
Near-infrared spectroscopy probe and channel settings.

We measured changes in the concentrations of oxygenated- (oxy-Hb) and deoxygenated-hemoglobin (deoxy-Hb) using a multichannel NIRS system (ETG-7000, Hitachi Medical Co., Japan). The emission probe (2 mm diameter) emitted near-infrared light with wavelengths of ~780 and 830 nm. Reflection of the infrared light was measured. The sampling rate was 10 Hz. Channels were arranged in a 3 by 5 rectangular lattice (22 channels) and kept in a silicon holder were placed in the frontal area for infants (see Figure 3). The bottom line of probes corresponds to the T3-Fp1-Fp2-T4 line according to the international 10–20 electrode system (36). The vertical midline of the channels was centered in the nasion-inion line. The emission and detection probe distance were set to 2 cm.

### Data Analysis

#### Behavior

Infants' looking responses to the target objects and the experimenter's eyes were both coded using a Behavior Coding System (PTS-113, DKH). Infants' gaze following in RJA trials were defined as following the experimenter's gaze to the object and fixating on the target object during the object presentation period. Gaze shifts in IJA trials were defined as alternately looking from the target object to the experimenter's eyes during the object presentation period. For this behavior to be coded, an infant had to shift their gaze from the target objects to the experimenter's eyes and then back again to the target. When the infants' gaze was diverted from the target object for more than 2 s, the trial was excluded from the analysis. Number of trials conducted, number of trials with infants' gaze following in RJA condition, and infants' gaze shift in IJA condition, and percentage of trials with joint attention episode were calculated for each infant (see Table 1). RJA trials without gaze following (26.4%) as well as IJA trials without gaze shifts (21.7%) were excluded. There was no significant difference in the number of trials with joint attention episode between RJA and IJA conditions, *t*_(10)_ = 0.27, *p* = 0.80.

**Table 1:** Number of trials conducted, number of trials with joint attention, and percentage of trials with joint attention episode.

	***N*** **of trials conducted**		***N*** **of trials with join attention**		**Percentage of trials with joint attention (%)**	
**Participants**	**RJA**	**IJA**	**RJA**	**IJA**	**RJA**	**IJA**
1	6	4	2	4	33.3	100.0
2	6	5	4	5	66.7	100.0
3	6	5	4	3	66.7	60.0
4	5	5	3	4	60.0	80.0
5	3	3	2	2	66.7	66.7
6	4	5	3	2	75.0	40.0
7	3	4	3	3	100.0	75.0
8	6	5	4	2	66.7	40.0
9	4	4	4	4	100.0	100.0
10	4	4	4	4	100.0	100.0
11	4	4	3	4	75.0	100.0
*M*	4.6	4.4	3.3	3.4	73.6	78.3
*SD*	1.2	0.7	0.8	1.0	20.2	24.0

#### NIRS Data

##### Activation

Infants' brain activations were measured using an event-related design. Concentration changes in oxygenated hemoglobin (oxy-Hb) are more strongly correlated with BOLD signals than those for deoxygenated hemoglobin (deoxy-Hb) (37–42). Therefore, our analysis focused on hemodynamic changes reflected in oxy-Hb. The oxy-Hb data were preprocessed using the plug-in-based analysis software Open PoTATo (43–45) written for Matlab (The MathWorks, Inc.). The raw data of oxy-Hb from individual channels were digitally high-pass-filtered at 0.0278 Hz to remove artifacts originating from task design or systematic fluctuations (41, 46). Trials with motion artifacts (signal variations <2 standard deviations from the mean over 0.3 s) were excluded. In addition, the remaining 2–5 trials were averaged and smoothed with a 5 s moving average. Then the original event markers locked to the onset of RJA and IJA trials were shifted to a time-point at the onset of joint attention episodes using behavioral data. The onset of an episode of joint attention is the onset of a situation in which the infant and the experimenter are looking at the presented stimuli simultaneously in both RJA and IJA trials. In the RJA trial, the onset of the joint attention episode can be defined as the time point when the infant followed the experimenter's gaze and fixated on the target object. In IJA trials, the onset of the joint attention episode is defined as the time point when the infant shifted their gaze from the experimenter's eyes to the object, and then the experimenter begins to fixate on the object. Supplementally, we also examined the effects of stimulus object presentation on infants' hemodynamic responses using hemodynamic changes that were time-locked to stimulus presentation.

The mean number of experimental trials conducted was 4.6 (*SD* = 1.2, range = 3–6) for the RJA condition and 4.4 (*SD* = 0.7, range = 3–5) for the IJA condition. The number of trials in which joint attention occurred was 3.3 trials, on average (*SD* = 0.79, range = 2–4) for the RJA condition and 3.4 trials (*SD* = 1.03, range = 2–5) for the IJA condition. For both RJA and IJA conditions, changes in concentration of oxy-Hb were analyzed for two different temporal windows at 5–10 s (Time Window 1) and 10–15 s (Time Window 2) after the onset of a joint attention episode. These two time windows were selected to examine changes in infant's brain activity in response to two different components of joint attention. In Time Window 1, infants' brain activity in response to joint attention episodes in which the adult and child were looking at the same target were examined. In Time Window 2, the infants' brain activity in response to mutual interaction where the adult turned toward the child and mentioned the target was also examined. Although it is difficult to strictly separate the brain responses of these two components, we explanatory divided the joint attention episodes into two time windows, the first half (Time window 1) and the second half (Time window 2). Data from the first 5 s after the onset of a joint attention episode were excluded to avoid transition period effects. In addition, the mean change in concentration of oxy-Hb was calculated over a span of 5 s before the onset of each trial, which was considered as baseline.

The statistical significance of differences between baseline hemodynamic changes and those observed in the two joint attention conditions were determined for each channel using a two-tailed paired-sample *t*-test. Paired-sample *t*-tests were also conducted for each channel to directly compare the hemodynamic changes observed in the RJA and IJA conditions. In addition, the brain regions underlying each activated channel were estimated using the virtual registration method for NIRS channels (47).

### Functional Connectivity

The method for calculating the task-based functional connectivity of brain activity in the RJA and IJA conditions was similar to that of previous studies examining functional connectivity in infants using fNIRS (48, 49). First, for each infant, the Matlab function “corrcoef” (The MathWorks Inc., Natick, MA, USA) was used to calculate the pairwise correlation coefficient (*r*) between the averaged time course of oxy-Hb in all 22 measurement channels. The correlation coefficients (*r*) were then subjected to Fisher's z-transformation [*z* (*r*)] to make the statistical distribution of correlation coefficients for each condition close to a normal distribution. Individual *z* (*r*) values in each of the RJA or IJA conditions were examined using a one-sample *t*-test against zero for the measurement channels. The calculated *t-*values were transformed into *z*-statistics according to the equation z = (t - t)/σ (t and σ represent the mean and SD, respectively). Functional connectivity between all channel pairs (22 × 21 pairs) except the same channel pair were examined. To compare the functional connectivity between RJA and IJA conditions, the z-values were subjected to paired-sample *t*-test for the channels that showed significant functional connectivity in each condition.

## Results

### Behavioral Results

Overall, gaze following by infants occurred in 73.6% of RJA trials (*SD* = 20.2) and infants' initiations of an adult's gaze shift occurred in 78.3% of IJA trials (*SD* = 24.0). These gaze-following results were consistent with those reported by Striano and Stahl (50).

### NIRS Results

#### Activation

Of the 22 infants, 11 were excluded from the final statistical analysis for: (a) refusal to wear the NIRS probe (*n* = 2), (b) failure to obtain more than two usable trials per condition, due to excessive motion artifacts (*n* = 7), (c) bad probe attachment due to hair obstruction (*n* = 1), or (d) no occurrence of gaze shifts (*n* = 1). Although many participants did not meet our criteria for inclusion in the analysis, this attrition rate was within the range for previous fNIRS studies that measured awake infants (22, 51–53). Effect size (Cohen's *d*) and power were calculated with G^*^power 3 (54).

**Figure 4:**
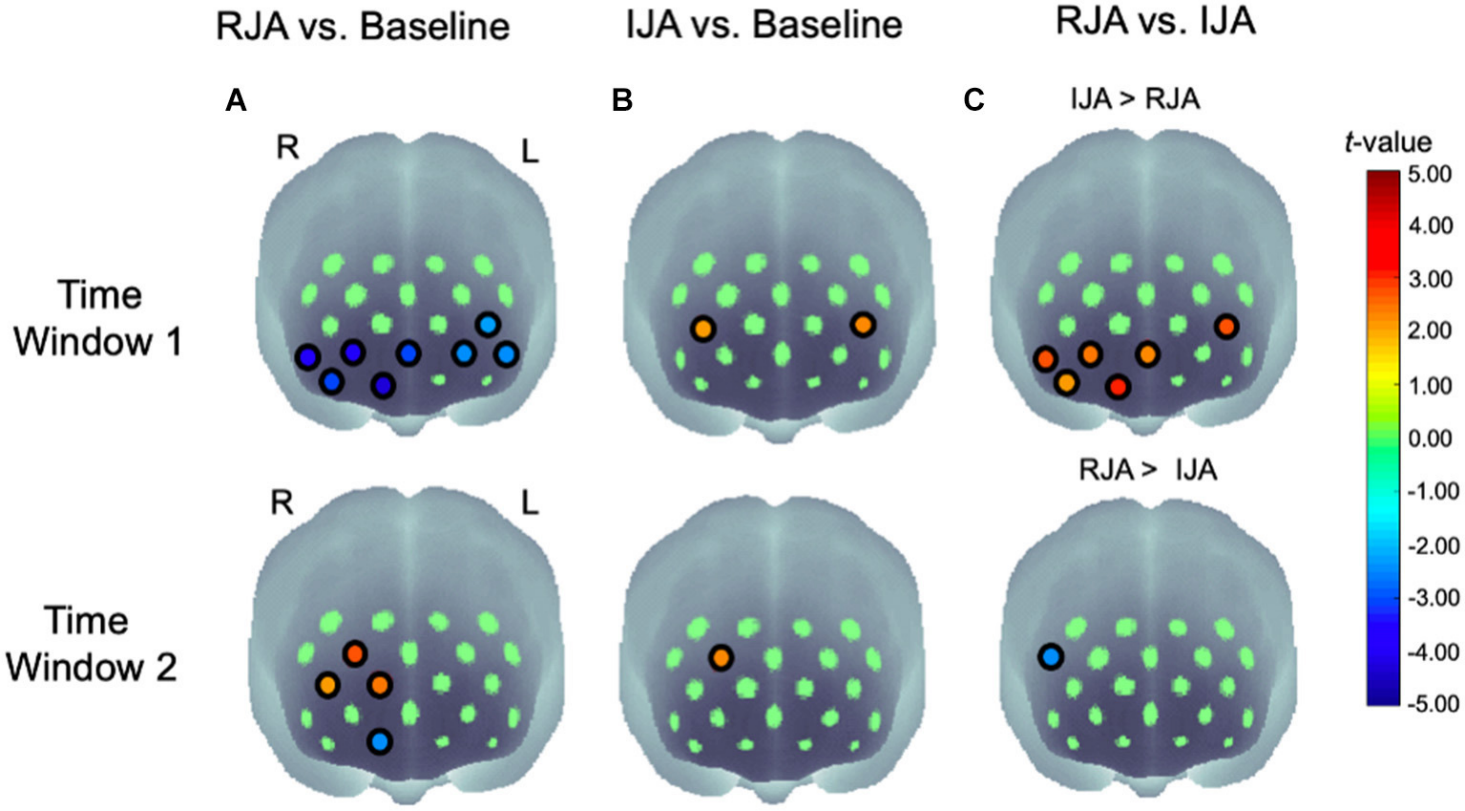
Statistical maps (*t*-maps) of oxy-Hb changes of **(A)** RJA vs. baseline, **(B)** IJA vs. baseline, and **(C)** RJA vs. IJA at 5–10 s (Time Window 1) and 10–15 s (Time Window 2) after the onset of the joint attention episode. The *t*-values for each channel are color coded as indicated by the color bar. L, left hemisphere; R, right hemisphere.

A paired-sample *t*-test against the baseline was conducted to examine the statistical significant differences between baseline hemodynamic changes and those observed in the two joint attention conditions for each channel (see Table 2; Figure 4).

**Table 2:** Statistical results for *t*-tests with comparison between he baseline and the RJA and IJA condition and then comparison with the RJA and IJA condition; the hemodynamic changes were time-locked to the onset of join attention episode.

	**Channel**	**Time window (s)**	***t* (10)**	***p* (uncorrected)**	***p* (FDR-corrected)**	***d***
RJA vs. baseline	3*	5–10	−4.39	0.001	0.030	−1.32
	3	10.15	−2.45	0.034	0.250	−0.74
	4	5–10	−3.00	0.013	0.059	−0.90
	5	5–10	−2.27	0.039	0.144	−0.71
	6	5–10	−2.34	0.041	0.130	−0.71
	7	5–10	−3.05	0.012	0.067	−0.92
	8*	5–10	−3.61	0.005	0.035	−1.09
	9*	5–10	−3.71	0.004	0.045	−1.12
	10	5–10	−2.23	0.050	0.144	−0.67
	12	10–15	2.52	0.031	0.337	0.76
	13	10–15	2.38	0.039	0.213	0.72
	17	10–15	2.95	0.015	0.319	0.89
IJA vs. baseline	10	5–10	2.74	0.021	0.462	0.83
	13	5–10	2.62	0.026	0.284	0.79
	17	10–15	2.32	0.043	0.946	0.70
IJA vs. RJA	3	5–10	3.06	0.012	0.270	0.97
	4	5–10	2.26	0.047	0.170	0.89
	7	5–10	2.45	0.034	0.150	0.75
	8	5–10	2.63	0.025	0.140	0.80
	9	5–10	2.83	0.018	0.200	0.87
	10	5–10	2.82	0.018	0.130	0.84
	18	10.15	−2.78	0.020	0.430	−0.84

*FDR-corrected p < 0.05.

**Figure 5:**
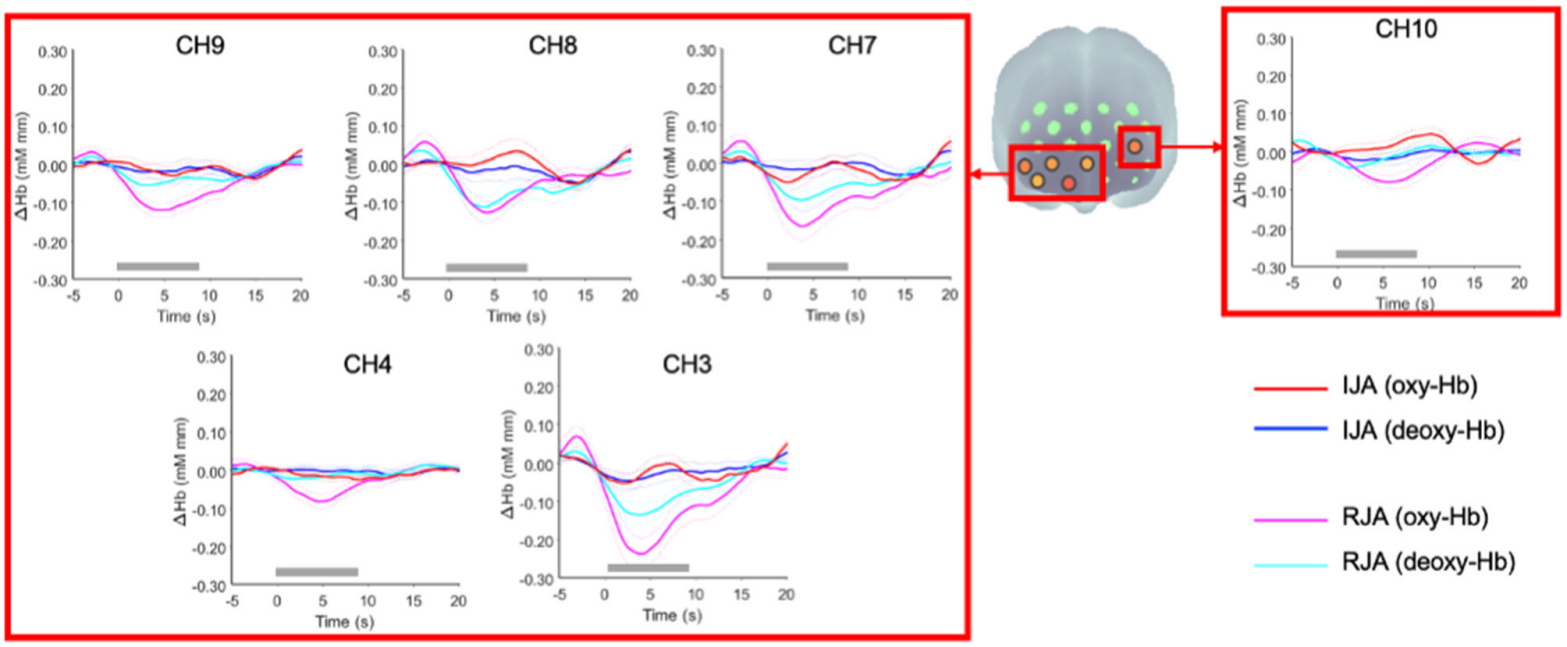
Grand-averaged time courses for changes in oxy-Hb and deoxy-Hb under IJA and RJA conditions in CH3, CH4, CH7, CH8, CH9, and CH10. The red and blue solid lines represent oxy-Hb and deoxy-Hb changes during the IJA condition, respectively. The magenta and cyan solid lines indicate oxy-Hb and deoxy-Hb during the RJA condition, respectively. The dashed lines represent ± 1 standard error of the mean (*SE*). Time 0 refers to the onset of joint attention episode. The thick gray lines represent the 8 s duration of the joint attention episode.

At the 5–10 s time window (Time Window 1), the results revealed that oxy-Hb concentration was significantly decreased in RJA condition at the uncorrected *p* < 0.05 level in CH3, CH4, CH5, CH6, CH7, CH8, CH9, and CH10 (Figure 4, top left panel). These analyses detected significant differences at an *FDR*-corrected *p* < 0.05 for CH3, CH8, and CH9. For IJA, a significant increase in oxy-Hb was observed in CH10 and CH13 at the uncorrected *p* < 0.05 level (Figure 4, top middle panel). These analyses detected no significant difference at an *FDR*-corrected *p* < 0.05. Next, RJA conditions were directly compared with IJA conditions using a two-tailed paired *t*-test for each channel (Figure 4 top right panel). A significant increase in the oxy-Hb was observed in CH3, CH4, CH7, CH8, and CH9, for the middle and right ventral prefrontal region and CH10 in the left dorsolateral regions under IJA conditions when compared with RJA conditions (see Figure 5 and grand-averaged time courses for changes in oxy-Hb and deoxy-Hb in all 21 channels are presented in Supplementary Figure). These analyses detected no significant difference at an *FDR*-corrected *p* < 0.05.

At the 10–15 s time window (Time Window 2), the results revealed that oxy-Hb was significantly decreased in RJA condition at the uncorrected *p* < 0.05 level in CH3. In addition, a significant increase in the oxy-Hb was observed in CH12, CH13, and CH17. These analyses detected no significant difference at an *FDR*-corrected *p* < 0.05 (Figure 4, bottom left panel). For IJA, a significant increase in oxy-Hb was observed in CH17 at the uncorrected *p* < 0.05 level (Figure 4, bottom middle panel). These analyses detected no significant difference at an *FDR*-corrected *p* < 0.05. When RJA conditions were directly compared with IJA conditions using a two-tailed paired *t*-test for each channel, a significant increase in oxy-Hb was observed in CH18 for the right dorsal prefrontal region under IJA conditions when compared to RJA conditions (Figure 4 bottom right panel).

We additionally examined the effect of presentation of stimuli on hemodynamic response in infants using the hemodynamic changes that were time-locked to the presentation of stimulus objects (see Supplementary Table). At the 5–10 s time window, a paired-sample *t*-test revealed that oxy-Hb concentration was significantly decreased in RJA condition when compared with the hemodynamic changes during the 5 s preceding the onset of the target presentation at the uncorrected *p* < 0.05 level in CH1, CH3, CH4, CH5, CH6, CH7, CH8, CH9, and CH10. These analyses detected significant differences at an *FDR*-corrected *p* < 0.05, for CH3, CH4, CH8, CH9, and CH10. In IJA, a significant decrease in oxy-Hb was also observed in CH2, CH3 CH8, and CH9 at the uncorrected *p* < 0.05 level. These analyses detected no significant difference at an *FDR*-corrected *p* < 0.05. When RJA conditions were directly compared with IJA conditions using a two-tailed paired *t*-test, we did not find any significant differences in the hemodynamic changes between the two conditions in any channel at the uncorrected *p* < 0.05 level.

At the 10–15 s time window, a paired-sample *t*-test revealed that oxy-Hb was significantly decreased in RJA condition when compared with the hemodynamic changes during the 5 s preceding the onset of the target presentation at the uncorrected *p* < 0.05 level in CH3, CH4, CH8, and CH9. These analyses detected no significant difference at an *FDR*-corrected *p* < 0.05 level. In IJA, a significant decrease in oxy-Hb was also observed in CH9 and CH10 at the uncorrected *p* < 0.05 level. These analyses detected no significant difference at an *FDR*-corrected *p* < 0.05. When RJA conditions were directly compared with IJA conditions using a two-tailed paired *t*-test, we did not find any significant differences in the hemodynamic changes between the two conditions in any channel at the uncorrected *p* < 0.05 level.

#### Functional Connectivity

**Figure 6:**
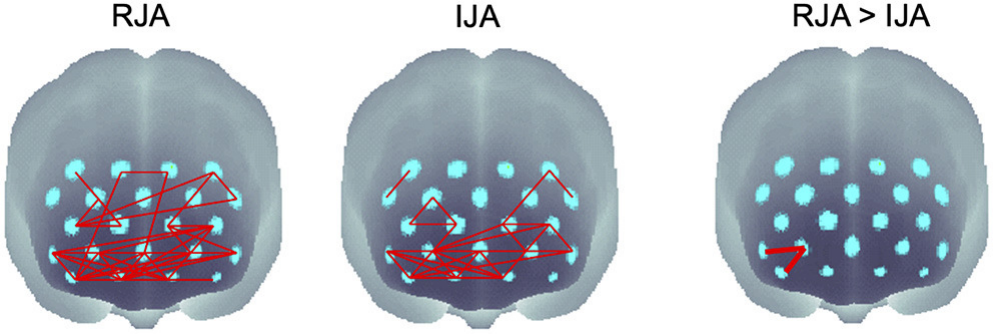
Spatial functional connectivity maps under the RJA condition, the IJA condition, and comparison with the RJA and IJA condition. Significant connections are shown by red lines for IJA and RJA conditions (uncorrected *p* < 0.01) and by thick red lines for the comparison between the RJA and IJA (*FDR*-corrected *p* < 0.05).

We assessed the spatial distribution of functional connectivity in frontal regions in two conditions, RJA and IJA. The results showed that the one-sample *t*-test of *z* (*r*) values in each of the two conditions (RJA and IJA) showed significantly different connectivity at the uncorrected *p* < 0.01 level (Figure 6). In both RJA and IJA, there were significantly higher correlations in a widely distributed area centered on the ventral frontal region and extending to the dorsal frontal region. When the functional connectivity between the RJA and IJA conditions was examined by paired sample *t*-test, the functional connectivity within the right ventral frontal region was significantly higher than that in the IJA condition (*FDR*-corrected *p* < 0.05).

## Discussion

In the current study, we examined cerebral hemodynamic responses and functional connectivity during joint attention episodes for typical infants using fNIRS. To examine the neural substrates of this in the context of real-time interactions between infants and adults, we developed a naturalistic face-to-face interaction paradigm. This live-interactive paradigm with fNIRS was successfully performed, which obtained task-specific cerebral activations as well as the behavioral results.

One component consists of deactivation of the bilateral prefrontal regions with the onset of joint attention episodes, which is observed during RJA conditions when compared to the baseline. The decreases were broader and more pronounced in the right ventral prefrontal cortex than in the left. This result was inconsistent with the finding of higher ventral prefrontal activation in the RJA condition compared to the IJA condition in an fMRI study using adult participants (14). However, interestingly, similar decreases in the oxy-Hb in the infant prefrontal regions have been reported for a variety of social stimuli. McDonald et al. (55) reported decreases in the oxy-Hb in the frontopolar and dorsolateral prefrontal regions in 6-month-old infants during 5–10 s after the presentation of non-verbal communicative sounds such as laughter. In addition, Xu et al. (56) also reported a decrease in the oxy-Hb in the infant's frontopolar, covering mPFC, dorsomedial, and dorsolateral prefrontal regions at 5–6 months of age during a peek-a-boo with an animated character or a female stranger presented on a monitor. Furthermore, Behrendt et al. (57) examined prefrontal activity in 7–9-month-old infants using the still-face paradigm during a live face-to-face interaction with their mothers. The results showed a decrease in the oxy-Hb in the middle frontal regions in response to their mothers' happy face when compared to a female stranger. In the same measurement channels, there was an increase in the oxy-Hb in response to a female stranger's happy face. In Grossmann and Johnson (22), who examined brain activity during joint attention, similar observations were also obtained in the more dorsal right prefrontal region. In the present study, the measurement channels with significant decreases in the oxy-Hb compared to baseline were regions similar to those reported in McDonald et al. (55), Xu et al. (56), and Behrendt et al. (57) with the frontopolar PFC, covering the mPFC and dorsolateral, as well as more ventral prefrontal regions. The mPFC is a part of the social cognitive network or default mode network, and its deactivation can be considered to reflect an infant's attention to the external world. Compared to IJA, RJA requires greater coordinated attention to another's gaze shift. Previous fNIRS studies also suggest that a higher attention load of visual stimuli decreases the oxy-Hb changes in the frontal area [e.g., (56, 58)]. When the hemodynamic changes were time-locked to the stimuli presentation, infants' hemodynamic responses in the prefrontal regions generally decreased in both RJA and IJA conditions (see Supplementary Table). Furthermore, previous infant studies also reported decreased activity to stimuli that were socially important to the infant (e.g., the mother's happy face). Taken together, we suggest that infants may have more attention for an object cued by an adult's joint attention cues during RJA than IJA.

The second component entails an increased bilateral prefrontal activation, which was observed in the IJA condition when compared to the baseline following the onset of a joint attention episode. In the IJA condition, significant increases in the oxy-Hb were observed in the right and middle ventral prefrontal areas, and left dorsolateral prefrontal area in direct comparison to the RJA condition. This result is partially consistent with a fMRI study in adults that found higher activation in the middle frontal cortex in the IJA condition compared to the RJA condition (14). This result also suggests that broader prefrontal regions are associated with IJA in older infants compared to Grossmann et al. (23), who demonstrated that a significant increase in the activation of the left prefrontal cortex occurs when engaged in IJA.

Grossmann and Johnson (22) observed a significant increase in the activation of the left dorsal prefrontal cortex when infants followed the adult's gaze shift. However, in the current study, we found decreased activity in the bilateral ventral prefrontal regions, followed by an increase in activation in the dorsolateral frontal cortex of the right hemisphere under RJA conditions. This difference may be attributed to the infants' age. In Grossmann and Johnson (22), 5-month-old infants were examined whereas 7–12-month-old infants participated in our study. In typical development IJA by gaze shift and pointing develops between 9 and 12 months of age. Before 9 months of age, infants engage in joint attention only by following other's gaze shifts and joint attention is mainly supported and maintained by the adult. After this time, the children begin to develop the spontaneous initiation of joint attention. The current data suggests that infants' spontaneous initiation of gaze shift to coordinate attention with others may influence infant hemodynamic activations in the bilateral dorsolateral frontal cortex. Dorsal attention networks are involved in the top-down control of endogenous attention (31). IJA requires volitional, goal-directed allocation of attention, which may be supported by the bilateral attention system.

In addition, IJA involves greater self-reference processing and the coordinated parallel processing of self and other-referenced information than RJA (59). Therefore, the increased frontal activation during IJA compared to RJA may partly be due to self-referenced processing in IJA. Indeed, the left prefrontal area, CH10, corresponds to the dorsomedial and dorsolateral prefrontal cortex in the previous fNIRS studies (56, 60) which is the central area for mental process (15, 61). Neural activity of these areas has been shown to be decreased when individuals engage in externally-focused tasks. On the other hand, the activations have been increased when they engage in internally-focused tasks involving self-referencing (62). As mentioned above, IJA involves coordinated processing of self and other-referenced information, which is a significant part of the mental process. IJA represents a base form of human communication. Using the paradigm which invoke endogenous motivation to share attention robustly, the present study reveals the early neural substrates for the mental functions underlying such communication.

Furthermore, in the present study, when the IJA and RJA conditions were directly compared, the left dorsolateral frontal cortex showed significantly higher activation in the IJA condition than in the RJA condition. Previous studies have indicated that left frontal cortex activation is related to the processing of positive emotion or approach motivation during interactions between adults and infants (22, 51, 53). For both conditions, infants and the experimenter shared visual attention with the object. However, under IJA conditions infants might be more endogenously motivated to share their attention to the objects with others compared to RJA conditions. This positive social motivation may be reflected by the highlighted activity in the left dorsal prefrontal region. Along with the results of the present study, this raises the possibility that increased left prefrontal responses during IJA may reflect the infant's processing of positive emotion by sharing attention on target objects with others.

In the present study, the brain functional connectivity during joint attention episode was also examined. Significant functional connectivity was found in a widely distributed area within the frontal lobe, whether joint attention episode was initiated by gaze shifts of adults or of infants. The key area of functional connectivity was the ventral frontal area, and significant connectivity was also found in the dorsal frontal regions. The results of functional connectivity showed significantly higher functional connectivity within the right ventral region in the RJA condition than in the IJA condition. There were no regions with higher functional connectivity in the IJA than in the RJA. Despite previous studies suggesting a greater contribution of the frontal lobe in the IJA than in the RJA [e.g., (63)], we did not find significantly higher functional connectivity in the IJA. In the RJA condition, the significant functional connectivity found within the right ventral frontal area may reflect a salient stimulus change in the environment, i.e., the facilitation of externally focused attention to the appearance of the target of joint attention. In contrast, in the IJA condition, infants were required to allocate their attention to the stimulus and the gaze of others by inhibiting their attention to the target objects. Thus, because of the inhibition of attention to the external environment, functional connectivity in this region is lower in the IJA than in the RJA.

The findings of the present study strongly suggest that infants are sensitive to RJA and IJA and recruit specific brain regions localized in the prefrontal cortex. Distinct regions include the right ventral prefrontal areas for RJA and the left dorsal prefrontal areas for IJA. Importantly, the dorsal prefrontal cortex engaged in mentalization was found to involve IJA processing in young infants.

In this study, fNIRS was used to measure brain functions in infants. Therefore, the limitation of this study is that only frontal cortical activity was measured and examined. Since it has been suggested that joint attention involves multiple systems distributed throughout the entire brain, including not only cortical but also subcortical regions, as well as temporal and parietal regions (63), further studies should also include these broader brain regions.

## Data Availability Statement

The dataset generated for this study are available, under the restriction of ethic permission obtained from our university, on request to the corresponding author.

## Ethics Statement

The studies involving human participants were reviewed and approved by Faculty of Literature, Keio University. Written informed consent to participate in this study was provided by the participants' legal guardian/next of kin. Written informed consent was obtained from the individual(s), and minor(s)' legal guardian/next of kin, for the publication of any potentially identifiable images or data included in this article.

## Author Contributions

NN, YM, and SK were responsible for design the experiments and analyzing the data. NN, YM, and J-iY were responsible for collecting the data. NN and YM were responsible for writing the article. All authors contributed to the article and approved the submitted version.

## Funding

This research was supported by JST, CREST Social Imaging and JSPS, KAKENHI Grants (16K21365 and 21K02360).

## Conflict of Interest

The authors declare that the research was conducted in the absence of any commercial or financial relationships that could be construed as a potential conflict of interest.

## Publisher's Note

All claims expressed in this article are solely those of the authors and do not necessarily represent those of their affiliated organizations, or those of the publisher, the editors and the reviewers. Any product that may be evaluated in this article, or claim that may be made by its manufacturer, is not guaranteed or endorsed by the publisher.

## Supplementary Material

Supplementary Figure Grand-averaged time courses for changes in oxy-Hb and deoxy-Hb under IJA and RJA conditions in all 22 channels. The red and blue solid lines represent oxy-Hb and deoxy-Hb changes during the IJA condition respectively. The magenta and cyan solid lines indicate oxy-Hb and deoxy-Hb during the RJA condition respectively. The dashed lines represent ± 1 standard error of the mean (*SE*). Time 0 refers to the onset of joint attention episode. The thick gray lines represent the 8 s duration of the joint attention episode.

Supplementary Table Statistical results for *t*-tests with comparison between the baseline and the RJA and IJA condition; the hemodynamic changes were time-locked to the stimuli presentation.
