# Supplementary figures and images for: Infants' Prefrontal Hemodynamic Responses and Functional Connectivity During Joint Attention in an Interactive-Live Setting
Source: Front Med Technol. 2022 Jun 15;4:821248. doi: 10.3389/fmedt.2022.821248 (PMC9240356; doi:10.3389/fmedt.2022.821248)

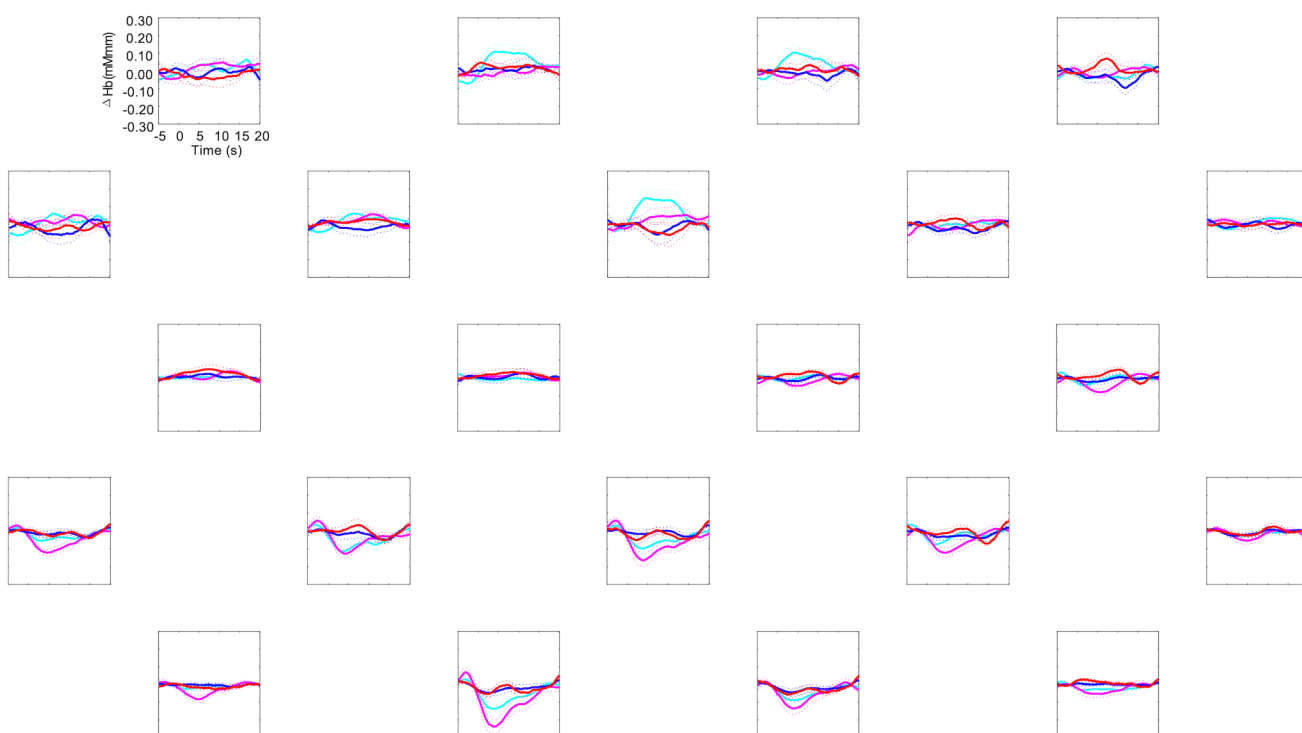

Supplement: Supplementary Figure — Grand-averaged time courses for changes in oxy-Hb and deoxy-Hb under IJA and RJA conditions in all 22 channels. The red and blue solid lines represent oxy-Hb and deoxy-Hb changes during the IJA condition respectively. The magenta and cyan solid lines indicate oxy-Hb and deoxy-Hb during the RJA condition respectively. The dashed lines represent ± 1 standard error of the mean (SE). Time 0 refers to the onset of joint attention episode. The thick gray lines represent the 8 s duration of the joint attention episode. [file Image_1.pdf]
