# Supplementary material for: Infants' Prefrontal Hemodynamic Responses and Functional Connectivity During Joint Attention in an Interactive-Live Setting
Source: Front Med Technol. 2022 Jun 15;4:821248. doi: 10.3389/fmedt.2022.821248 (PMC9240356; doi:10.3389/fmedt.2022.821248)
Supplement: Supplementary file 2 [file Table_1.docx]

Supplementary Table. Statistical results for t-tests with comparison between the baseline and the RJA and IJA condition; the hemodynamic changes were time-locked to the stimuli presentation.

Oxy	Channel	Time Window (s)	*t* (10)	*p* (uncorrected)	*p* (FDR-corrected)	*d*
RJA vs. Baseline	1	5-10	-5.41	0.039	0.094	-0.72
	3*	5-10	-4.53	0.001	0.012	-1.37
	3	10-15	-2.94	0.015	0.326	-0.89
	4*	5-10	-3.27	0.008	0.037	-0.98
	4	10-15	-2.61	0.026	0.189	-0.79
	5	5-10	-2.78	0.020	0.054	-0.84
	6	5-10	-2.78	0.019	0.061	-0.84
	7*	5-10	-3.17	0.010	0.037	-0.95
	8*	5-10	-4.28	0.002	0.012	-1.29
	8	10-15	-2.41	0.037	0.202	-0.73
	9*	5-10	-5.41	0.000	0.007	-1.63
	9	10-15	-2.66	0.024	0.265	-0.80
	10*	5-10	-4.00	0.003	0.014	-1.21
IJA vs. Baseline	2	5-10	-2.42	0.036	0.198	-0.73
	3	5-10	-3.11	0.011	0.121	-0.94
	8	5-10	-2.49	0.032	0.235	-0.75
	9	5-10	-3.31	0.008	0.174	-1.00
	9	10-15	-2.56	0.028	0.313	-0.77
	10	5-10	-2.39	0.038	0.167	-0.72
	10	10-15	-2.88	0.016	0.362	-0.87
